# Association between Striatal Subregions and Extrastriatal Regions in Dopamine D_1_ Receptor Expression: A Positron Emission Tomography Study

**DOI:** 10.1371/journal.pone.0049775

**Published:** 2012-11-21

**Authors:** Hironobu Fujiwara, Hiroshi Ito, Fumitoshi Kodaka, Yasuyuki Kimura, Harumasa Takano, Tetsuya Suhara

**Affiliations:** 1 Clinical Neuroimaging Team, Molecular Neuroimaging Program, Molecular Imaging Center, National Institute of Radiological Sciences, Chiba, Japan; 2 Biophysics Program, Molecular Imaging Center, National Institute of Radiological Sciences, Chiba, Japan; Centre for Addiction and Mental Health, Canada

## Abstract

The mesencephalic dopamine (DA) system is the main DA system related to affective and cognitive functions. The system consists of two different cell groups, A9 and A10, which originate from different regions of the midbrain. The striatum is the main input from the midbrain, and is functionally organized into associative, sensorimotor and limbic subdivisions. At present, there have been few studies investigating the associations of DA functions between striatal subdivisions and extrastriatal regions. The aim of this study was to investigate the relationship of DA D_1_ receptor (D_1_R) expression between striatal subdivisions and extrastriatal regions in humans using positron emission tomography (PET) with voxel-by-voxel whole brain analysis. The PET study was performed on 30 healthy subjects using [^11^C]SCH23390 to measure D_1_R expression. Parametric images of binding potentials (*BP*_ND_) were created using the simplified reference tissue model. Regions of interest were defined for striatal subdivisions. Multiple regression analysis was undertaken to determine extrastriatal regions that were associated with each striatal subdivision in *BP*_ND_ using statistical parametric mapping 5. The *BP*_ND_ values of associative, sensorimotor and limbic subdivisions were similarly correlated with those of multiple brain regions. Regarding the interrelationships among striatal subdivisions, mutual correlations were found among associative, sensorimotor and limbic subdivisions in *BP*_ND_ as well. The relationships in *BP*_ND_ between striatal subdivisions and extra-striatal regions suggest that differential striatal subdivisions and extrastriatal regions have a similar biological basis of D_1_R expression. Different DA projections from the midbrain did not explain the associations between striatal subdivisions and extrastriatal regions in D_1_R expression, and the DA-related neural networks among the midbrain, striatum and the other regions would contribute to a similar D_1_R expression pattern throughout the whole brain.

## Introduction

The mesencephalic dopamine (DA) system is the main DA system, and it is related to affective and cognitive functions such as reward processing. The system is roughly divided into different groups, A9 and A10, whose cells are located in different regions of the midbrain, the substantia nigra (SN) and the ventral tegmental area (VTA), respectively. These different projections have been reported in rats, monkeys and humans [1],[2],[3]. The striatum provides the main input from the midbrain. Histologically, this region is not uniform, and it is functionally divided into striatal subdivisions termed associative (AST), sensorimotor (SMST) and limbic (LST), which process information related to cognitive, sensorimotor, and emotional functions, respectively [4]. The concept is based on neural networks termed “Cortico-striatal-thalamo-cortical loops” [5]. In brief, functionally different networks between each striatal subdivision and extrastriatal regions would exist through dopaminergic, glutaminergic and gamma-butylic amino acid (GABA) neurotransmissions, and these neurotransmissions interact with each other [6]. Regarding DA projections, A9 would project to the dorsal striatum (AST and SMST) and A10 to LST. A10 would have direct projections to cortical regions as well.

Ample literature describes the differential DA pathways and the distribution of DA receptors by *in vitro* methods, including distinct DA projections from the midbrain to the dorsal and ventral striatum [7], region-by-region differences of DA receptor distribution in the cortex [8], and alterations of DA projections in several neuropsychiatric illnesses [9],[10]. Regarding neuroimaging studies, several reports have suggested the relationships between DA functions and cognitive functions [11],[12], and the association of DA functions with the pathophysiology of neuropsychiatric illnesses [10],[13]. Thus, it is worthwhile investigating DA functions in their relationships among different regions of the human brain by *in vivo* methods, especially between the DA receptor-rich regions (striatum) and other regions, which could provide new insights for studies of DA-related cognitive functions and pathophysiologies of neuropsychiatric disorders.

However, at present, there have been few neuroimaging studies that directly demonstrated the relationship between striatal subdivisions and extrastriatal (ie., cortical) regions, which also have DA projections from the midbrain in DA receptor expressions. Clarification of this issue would lead to a better understanding of DA functions in region-by-region relationships, considering the manner of DA projections from the midbrain and the distinction of their targets, that is, the most DA-rich region, the striatum and cortical regions. In this sense, one possibility might be that DA receptor expressions are regulated differentially according to their origin of DA projection.

However, very recently, one positron emission tomography (PET) study has suggested that there was no relationship between cortical DA D_2_ receptor (D_2_R) densities and those of striatal regions [14]. Regarding another dopamine receptor subtype, DA D_1_ receptor (D_1_R), Rieckmann et al. reported that subdivisional striatal D_1_R densities are similarly associated with those of multiple cortical regions, concluding that D_1_R expressions in striatal and extrastriatal regions are not regulated independently, despite DA projections from different midbrain areas. In their study [15], interregional association of D_1_R was assessed by a conventional method in terms of the analysis of PET images, that is, regions of interests (ROIs) were traced manually on each individual subject’s image without spatial normalization. This method is potentially advantageous in preserving the information of raw images, but the results may partially depend on the rater’s procedure. Thus, the conventional manual tracing method and another method, voxel-by-voxel analysis, could be expected to complement each other in respect to confirming their reliability and validity.

The aim of the present study was to investigate the relationship between striatal subdivisions and extrastriatal regions in DA D_1_ receptor (D_1_R) expression using PET in healthy humans by voxel-by-voxel analysis, a potentially more objective method than the manual ROI-tracing method used by Rieckmann et al. [15]. We hypothesized that D_1_R availability of the striatum would be associated with the availability of extrastriatal regions regardless of its differential subdivisions, i.e., the D_1_R expressions of AST, SMST and LST would be similarly correlated with the expressions of extrastriatal regions.

## Methods

### Ethics Statement

In accordance with the Helsinki Declaration of Human Rights (2000), written informed consent was obtained from all volunteers after detailed explanation of the study. This study protocol was approved by the Ethics and Radiation Safety Committees of the National Institute of Radiological Sciences, Chiba, Japan.

### Subjects

A total of 30 healthy men (age = 25.4±5.9 [mean ± SD]) were recruited, and they gave their written informed consent for participation in this study. The subjects were free of somatic, neurological or psychiatric disorders on the basis of their medical history and magnetic resonance imaging (MRI) of the brain. They had no history of current or previous drug abuse.

### Pet Procedures

The PET system ECAT EXACT HR+(CTI-Siemens, Knoxville, TN) was used for all PET studies. The system provides 63 planes with a 15.5 cm axial field of view. The intrinsic spatial resolution was 4.3 mm in-plane and 4.2 mm full-width at half maximum (FWHM) axially. With a Hanning filter (cut-off frequency: 0.4 cycle/pixel), the reconstruct in-plane resolution was 7.5 mm FWHM. Data were acquired in three-dimensional mode. Scatter was corrected [16]. A head fixation device with thermoplastic attachments for individual fit minimized head movement during PET measurements. A 10-min transmission scan using a ^68^Ge- ^68^Ga line source was performed for correction of attenuation. After intravenous rapid bolus injection of [^11^C]SCH23390, data were acquired for 60 min in a consecutive series of time frames. The frame sequences consisted of thirty 2-min frames. Injected radioactivity was 197–235 MBq and specific radioactivity was 23–81 GBq/µmol at the time of injection.

### Mri Procedures

All MRI scanning was performed with a 1.5-T MR scanner (Philips Medical Systems, Best, The Netherlands). Three-dimensional volumetric acquisition of a T1-weighted gradient echo sequence produced a gapless series of thin transverse sections (TE: 9.2 ms; TR: 21 ms; flip angle: 30°; field of view: 256 mm; acquisition matrix: 256×256; slice thickness: 1 mm).

### Calculation Of Parametric Images

We used PMOD 3.1 software (PMOD Technologies Ltd., Zurich, Switzerland) for all the steps of the image processing and analysis. All MR images were coregistered to the PET images. MR images were transformed into standard brain size and shape by linear and non-linear parameters (anatomic standardization). The brain templates for anatomic standardization were Montreal Neurological Institute (MNI)/International Consortium for Brain Mapping (ICBM) 152 T1 templates as supplied with the PMOD software. All PET images were also transformed into standard brain size and shape by using the same parameters as for the MR images. Thus, brain images of all 30 subjects had the same anatomic format.

Binding potentials (*BP*_ND_) were calculated by the reference tissue model method on a voxel-by-voxel basis [17],[18]. *BP*_ND_ refers to the ratio of specifically bound radioligand to that of nondisplaceable radioligand in tissue at equilibrium. *BP*_ND_ is the typical measurement from reference tissue methods, as it compares the concentration of radioligand in receptor-rich to receptor-free regions [19]. In this study, parametric images, in which each voxel has its own *BP*_ND_ value, were generated using the cerebellum, a receptor-free region, as reference tissue.

### Data Analysis

**Figure 1:**
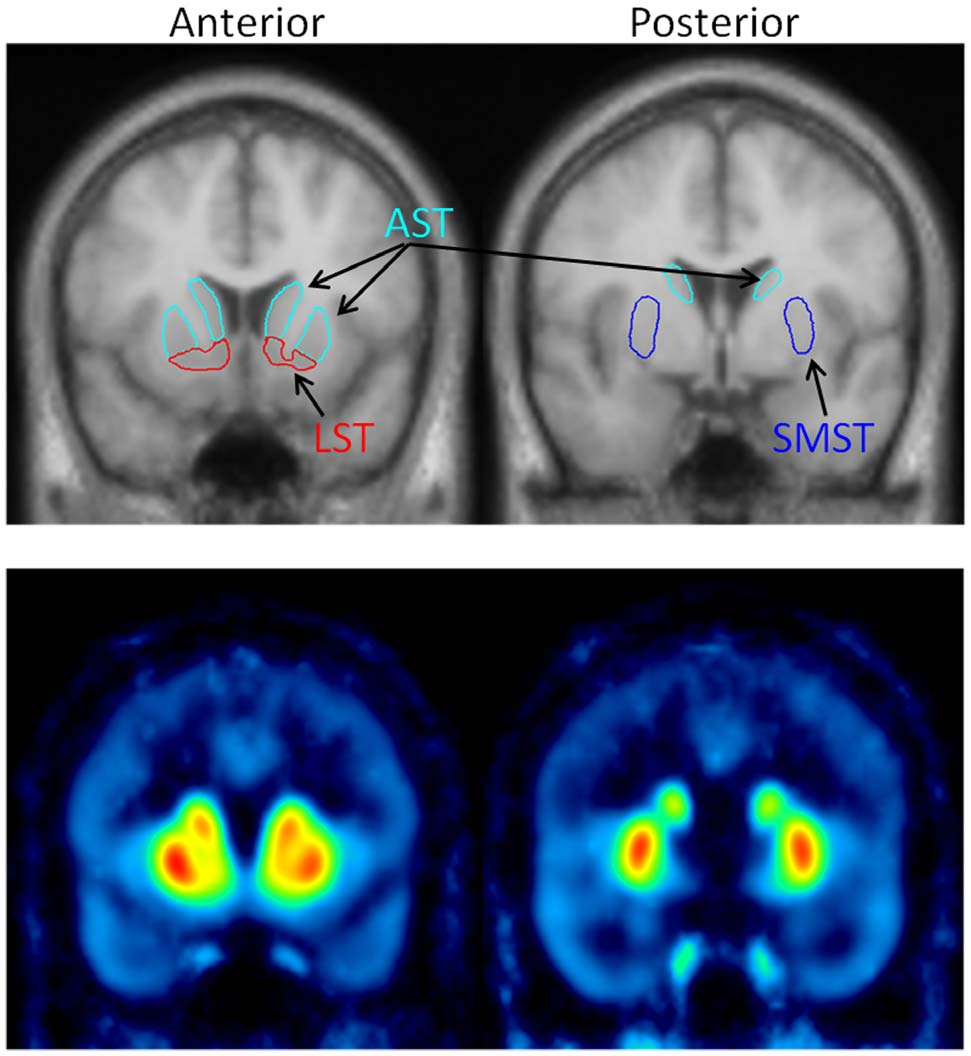
Definition of striatal functional subdivisions. Upper panel: MR images and regions of interest. Lower panel: parametric images corresponding to MR images in the upper panel.

ROIs were drawn on a standardized and averaged MR image of all the subjects, and this ROI object map was applied to the parametric images of each of the 30 subjects, that is, only one ROI object map was applied to the parametric images of each of the 30 subjects completely in the same manner. Thus, this method is a more objective way to measure BP_ND_ values than that with different ROIs for each subject. Boundaries for ROIs of striatal functional subdivisions were defined for each striatal subregion [20],[21]. The definition of the “functional subdivisions” was as follows: AST consisted of the precommissural dorsal caudate, precommissural dorsal putamen, and postcommissural caudate, and the *BP*_ND_ values of AST were calculated as the spatially weighted average of these three subregions; SMST and LST corresponded to the postcommissural putamen and ventral striatum, respectively (Figure 1).

Regarding the statistics, multiple regression analysis was performed by statistical parametric mapping (SPM5, Wellcome Trust Centre for Neuroimaging, London, UK) on a voxel-by-voxel basis after the BP_ND_ values of the striatal subdivisions were obtained. The values of each striatal subdivision were used as covariates of interest in the design matrix to determine the regions correlating with each striatal subdivision in terms of their D_1_R expression. Statistical thresholds were as follows: false recovery rate (FDR) p<0.05, extent threshold = 100 voxels. The results of the correlation were visualized in statistical parametric maps.

To confirm the result of voxel-by-voxel analysis with SPM5, Pearson’s correlation coefficient was also calculated using the actual BP_ND_ values in extrastriatal regions with SPSS version 18.0. The ROIs of extrastriatal regions included thalamic, cingulate, prefrontal, temporal and occipital regions, and the boundaries for the ROIs were based on previous reports [21],[22].

## Results

The *BP*_ND_ values of AST, SMST, and LST were 1.61±0.26, 1.70±0.24, and 1.36±0.19, respectively. The values were quite similar to our previous data for measuring D_1_R in the striatum [22].

**Figure 2:**
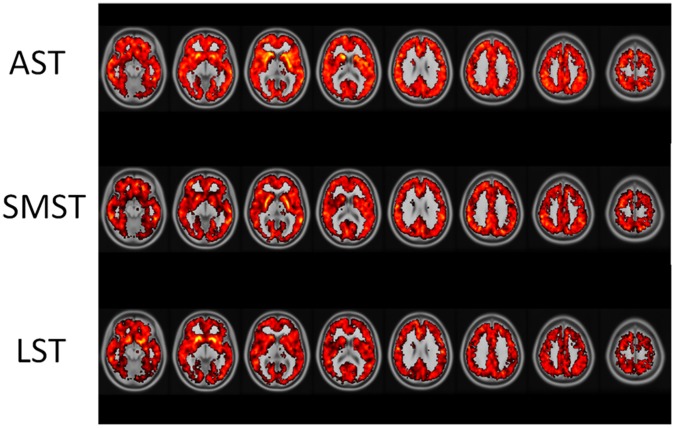
Correlation map of striatal functional subdivisions and extrastriatal regions, in addition to that of within striatal subdivisions. Correlations in brighter color (yellow) represent higher ones in terms of magnitude than those in red.

By voxel-by-voxel analysis, the values of each striatal subdivision (i.e., AST, SMST and LST) were positively correlated with those of multiple brain regions, i.e., frontal, temporal, parietal and occipital regions in a similar manner (Figure 2). Regarding the interrelationships among striatal subdivisions, mutual positive correlation was found among AST, SMST and LST in D_1_R *BP*_ND_ (Figure 2).

**Figure 3:**
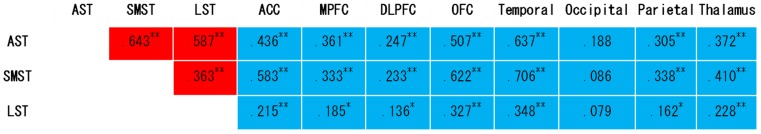
Correlations between striatum and extrastriatal regions and intercorrelations among striatal subdivisions in dopamine D_1_ receptor BP_ND_. **P<0.01, *P<0.05. Correlations in red: intercorrelations among striatal subdivisions. Correlations in blue: correlations between striatal subdivisions and extrastriatal regions. R^2^ values are presented for the correlations.

In addition, the interregional positive correlations in *BP*_ND_ were revealed to be significant by the ROI analysis, that is, by the analysis using SPSS software with the actual *BP*_ND_ values of each ROI (Figure 3).

## Discussion

The critical role of the DA system in cognitive functions has been suggested repeatedly, and abnormalities of the system have also been implicated in the pathophysiology of several neuropsychiatric disorders such as schizophrenia [13],[23] and Parkinson’s disease [10]. The main focus of those findings was restricted exclusively to D_1_R functions or abnormalities in terms of their expression levels, distribution, and localization in several brain regions. In this sense, there is little evidence that refers to a direct association of a DA-rich region (the striatum) and extrastriatal regions, where DA receptors are present. It has been suggested that cognitive functions such as executive function would be associated with the manner of interregional relationship in D_1_R expression [15], and therefore it would be worthwhile investigating the interregional patterns of bindings for studies of neuropsychiatric disorders in which cognitive dysfunction based on DA system dysregulation is considered to exist, such as schizophrenia and Parkinson’s disease.

We could replicate the findings of a previous study by Rieckmann et al. [15] that demonstrated the association of striatal subdivisions and cortices by a conventional manual tracing method. The major findings of the present study were as follows: (a) BP_ND_ values of all striatal subdivisions (i.e., AST, SMST and LST) were significantly correlated with those of multiple brain regions on a voxel-by-voxel basis: (b) regarding the interrelationships among striatal subdivisions, they were also mutually correlated in their BP_ND_ values.

These results suggest that D_1_R expressions in striatal subdivisions and extrastriatal regions are regulated uniformly. This could be explained by the complex connections of DA pathways throughout the whole brain. DA innervations from VTA and SN differentially project to striatal subdivisions as well as the cortical regions through A9 and A10, whereas glutaminergic innervations from cortical regions project to both VTA and SN via the striatum. Regarding the connection of the midbrain with the striatum, the midbrain has reciprocal projections both to (DA) and from (GABA) the striatum, with overlapping. Thus, DA pathways are connected via these pathways [4],[24], and this would lead to similarity of the regulation of D_1_R expressions among multiple brain areas, although differential DA projection from the midbrain (i.e., A9 and A10) is a part of the DA-related neural network.

Furthermore, striatal outputs to the cortex, which are altered by D_1_R stimulation/blockade, would affect immediate-early gene expressions such as c-fos expression (as functional markers) in cortical regions [25]. If the D_1_R function in each striatal subdivision uniformly affects the expressions in cortical regions, each striatal subdivision and the cortical regions are mutually correlated in a similar manner. In this study, interrelationships among striatal subdivisions were found in their BP_ND_ values, thus providing a convincing explanation for the uniform relationships between different striatal subdivisions and cortical regions in their D_1_R expression.

Finally, D_1_R expressions throughout the whole brain might be generally (genetically) associated with each other, i.e., the larger the expressions in the striatum, the larger in the other regions as well. However, further investigations including postmortem and animal studies would be needed to clarify the genetic influence on the D_1_R expression throughout the whole brain.

Several limitations need to be pointed out in the current study. First, the age of the participants was restricted to a relatively younger generation. Second, the subjects were all males. Further study with both male and female participants of a wider age range will be needed to give the findings a more generalized significance. Third, the reference tissue model has a potential limitation in terms of its theory, the assumption of the same non-specific binding throughout the whole brain, which might lead to a systemic bias in respect to between-region correlations of the bindings. To our knowledge, there has been no study that suggested a higher variation of receptor density corresponding to *BP*_ND_ in different regions compared with that of non-specific binding in the human population. However, the non-specific binding in tissue has been reported to be generally constant across species including humans [26]. Thus, the correlation of the current study would reflect the relationship in receptor density in itself rather than inter-individual variations of non-specific binding. Further theoretical and methodological improvements would be needed to assess interregional correlations of binding potentials more accurately, considering the influence of inter-individual variations in non-specific binding. Fourth, it could be argued that the interregional associations in the bindings in the current study may reflect the association in the serotonergic system in addition to the DA system because of the affinity of SCH23390 to 5-HT_2A_ receptors in cortical regions. However, this confounding effect of cortical 5-HT_2A_ binding would not be so critical in terms of the analysis of striatal and extrastriatal correlations because striatal binding reflects D_1_R density only, whereas the bindings of cortical regions are significantly confounded by 5-HT_2A_ receptors [15]. In the present study, the *BP*_ND_ values of striatal and extrastriatal regions were highly correlated, and thus the correlations are not always considered to represent different receptor associations. Finally, in general, completely accurate image processing (namely, coregistration and normalization) is difficult in voxel-based analyses. In this study, the accuracy of image processing and ROI adjustment on parametric images was confirmed by visual inspection for each subject. However, at present, there is no absolute procedure in this regard because of the variation of the individual’s brain in respect to its shape, size and sulcal anomaly. Further improvements in image processing technique would be necessary to raise the reliability of voxel-wise analysis.

### Conclusions

In conclusion, differential striatal functional subdivisions could be associated with cortical regions in terms of D_1_R expression in a similar manner. Although DA cell projections from VTA and SN innervate the striatum and extrastriatal regions via different DA pathways, DA-related neural networks throughout the whole brain including both striato-midbrain and cortical-striato connections would contribute to the association of the striatal subdivisions and extrastriatal regions in D_1_R expression. Further study will be needed to clarify the mechanisms of D_1_R expression regarding the interactions between DA and the other neurotransmitter systems such as glutamate, serotonin and GABA, and the mechanisms at molecular and genetic levels in the respective brain regions.

## References

[1] Dahlström A, Fuxe K (1964) A method for the demonstration of monoamine-containing nerve fibres in the central nervous system. Acta Physiol Scand 60: 293–4. 14131843 10.1111/j.1748-1716.1964.tb02891.x

[2] Di Carlo FJ, Melgar MD, Haynes LJ, Crew MC (1973) Metabolic profile of a new immunosuppressive agent, oxisuran: binding, RE stimulation, drug interaction. J Reticuloendothel Soc 14(4): 387–97. 4201612

[3] Felten DL, Sladek JR Jr (1983) Monoamine distribution in primate brain V. Monoaminergic nuclei: anatomy, pathways and local organization. Brain Res Bull 10: 171–284. 6839182 10.1016/0361-9230(83)90045-x

[4] Joel D, Weiner I (2000) The connections of the dopaminergic system with the striatum in rats and primates: an analysis with respect to the functional and compartmental organization of the striatum. Neuroscience 96: 451–74. 10717427 10.1016/s0306-4522(99)00575-8

[5] Alexander GE, Crutcher MD (1990) Functional architecture of basal ganglia circuits: neural substrates of parallel processing. Trends Neurosci 13: 266–71. 1695401 10.1016/0166-2236(90)90107-l

[6] Carlsson A, Waters N, Carlsson ML (1999) Neurotransmitter interactions in schizophrenia-therapeutic implications. Eur Arch Psychiatry Clin Neurosci 249: 37–43. 10.1007/pl00014183 10654107

[7] Haber SN, McFarland NR (1999) The concept of the ventral striatum in nonhuman primates. Ann N Y Acad Sci 877: 33–48. 10415641 10.1111/j.1749-6632.1999.tb09259.x

[8] Hurd YL, Suzuki M, Sedvall GC (2001) D1 and D2 dopamine receptor mRNA expression in whole hemisphere sections of the human brain. J Chem Neuroanat 22: 127–37. 11470560 10.1016/s0891-0618(01)00122-3

[9] Grace AA (1991) Phasic versus tonic dopamine release and the modulation of dopamine system responsivity: a hypothesis for the etiology of schizophrenia. Neuroscience 41: 1–24. 1676137 10.1016/0306-4522(91)90196-u

[10] Gaspar P, Duyckaerts C, Alvarez C, Javoy-Agid F, Berger B (1991) Alterations of dopaminergic and noradrenergic innervations in motor cortex in Parkinson’s disease. Ann Neurol 30: 365–74. 1683212 10.1002/ana.410300308

[11] Takahashi H, Kato M, Takano H, Arakawa R, Okumura M, et al (2008) Differential contributions of prefrontal and hippocampal dopamine D1 and D2 receptors in human cognitive functions. J Neurosci 28: 12032–12038. 19005068 10.1523/JNEUROSCI.3446-08.2008 PMC6671660

[12] Takahashi H, Matsui H, Camerer C, Takano H, Kodaka F, et al (2010) Dopamine D_1_ receptors and nonlinear probability weighting in risky choice.J Neurosci. 30: 16567–72. 10.1523/JNEUROSCI.3933-10.2010 PMC6634867 21147996

[13] Suhara T, Okubo Y, Yasuno F, Sudo Y, Inoue M, et al (2002) Decreased dopamine D2 receptor binding in the anterior cingulate cortex in schizophrenia. Arch Gen Psychiatry 59: 25–30. 11779278 10.1001/archpsyc.59.1.25

[14] Cervenka S, Varron A, Fransen E, Halldin C, Farde L (2010) PET studies of D2-receptor binding in striatal and extrastriatal brain regions: biochemical support in vivo for separate dopaminergic systems in humans. Synapse 64: 478–85. 20175222 10.1002/syn.20765

[15] Rieckmann A, Karlsson S, Larlsson P, Brehmer Y, Fischer H, et al (2011) Dopamine D1 receptor associations within and between dopaminergic pathways in younger and elderly adults: link to cognitive performance. Cereb Cortex 21: 2023–32. 21258043 10.1093/cercor/bhq266

[16] Watson CC, Newport D, Casey ME (1996) A single scatter simulation technique for scatter correction in 3D PET. In: Grangeat P, Amans JL (Eds.), Three-Dimensional Image Reconstruction in Radiology and Nuclear Medicine. Kluwer Academic Publishers, Dordrecht, The Netherlands, 255–268.

[17] Gunn RN, Lammertsma AA, Hume SP, Cunningham VJ (1997) Parametric imaging of ligand-receptor binding in PET using a simplified reference region model. NeuroImage 6: 279–87. 9417971 10.1006/nimg.1997.0303

[18] Lammertsma AA, Hume SP, 1996. Simplified reference tissue model for PET receptor studies. NeuroImage 4, 153–158. 10.1006/nimg.1996.0066 9345505

[19] Innis RB, Cunningham VJ, Delforge J, Fujita M, Gjedde A, et al (2007) Consensus nomenclature for in vivo imaging of reversibly binding radioligands. J Cereb Blood Flow Metab 27: 1533–9. 17519979 10.1038/sj.jcbfm.9600493

[20] Martinez D, Slifstein M, Broft A, Mawlawi O, Hwang DR, et al (2003) Imaging Human mesolimbic dopamine transmission with positron emission tomography. part II: amphetamine-induced dopamine release in the functional subdivisions of the striatum. J Cereb Blood Flow Metab 23: 285–300. 12621304 10.1097/01.WCB.0000048520.34839.1A

[21] Abi-Dargham A, Mawlawi O, Lombard L, Gil R, Martinez D, et al (2002) Prefrontal Dopamine D1 Receptors and Working Memory in Schizophrenia. J Neurosci 22: 3708–19. 11978847 10.1523/JNEUROSCI.22-09-03708.2002 PMC6758376

[22] Ito H, Takahashi H, Arakawa R, Takano H, Suhata T (2008) Normal database of dopaminergic neurotransmission system in human brain measured by postron emission tomography. NeuroImage 39: 555–565. 17962043 10.1016/j.neuroimage.2007.09.011

[23] Okubo Y, Suhara T, Suzuki K, Kobayashi K, Inoue O, et al (1997) Decreased prefrontal dopamine D1 receptors in schizophrenia revealed by PET. Nature 385(6617): 634–6. 9024661 10.1038/385634a0

[24] Haber SN, Fudge JL, McFarland NR (2000) Striatonigrostriatal pathways in primates form an ascending spiral from the shell to the dorsolateral striatum. J Neurosci 20: 2369–82. 10704511 10.1523/JNEUROSCI.20-06-02369.2000 PMC6772499

[25] Steiner H, Kitai ST (2000) Regulation of rat cortex function by D1 dopamine receptors in the striatum. J Neurosci 20: 5449–60. 10884328 10.1523/JNEUROSCI.20-14-05449.2000 PMC6772329

[26] Summerfield SG, Lucas AJ, Porter RA, Jeffrey P, Gunn RN, et al (2008) Toward an improved prediction of human in vivo brain penetration. Xenobiotica 38: 1518–35. 18979396 10.1080/00498250802499459

